# Predicting demographic characteristics from anterior segment OCT images with deep learning: A study protocol

**DOI:** 10.1371/journal.pone.0270493

**Published:** 2022-08-11

**Authors:** Yun Jeong Lee, Sukkyu Sun, Young Kook Kim

**Affiliations:** 1 Department of Ophthalmology, Seoul National University Hospital, Seoul, Korea; 2 Biomedical Research Institute, Seoul National University Hospital, Seoul, Korea; 3 Department of Ophthalmology, Seoul National University College of Medicine, Seoul, Korea; University of Toronto, CANADA

## Abstract

**Introduction:**

Anterior segment optical coherence tomography (AS-OCT) is a non-contact, rapid, and high-resolution *in vivo* modality for imaging of the eyeball’s anterior segment structures. Because progressive anterior segment deformation is a hallmark of certain eye diseases such as angle-closure glaucoma, identification of AS-OCT structural changes over time is fundamental to their diagnosis and monitoring. Detection of pathologic damage, however, relies on the ability to differentiate it from normal, age-related structural changes.

**Methods and analysis:**

This proposed large-scale, retrospective cross-sectional study will determine whether demographic characteristics including age can be predicted from deep learning analysis of AS-OCT images; it will also assess the importance of specific anterior segment areas of the eyeball to the prediction. We plan to extract, from SUPREME^®^, a clinical data warehouse (CDW) of Seoul National University Hospital (SNUH; Seoul, South Korea), a list of patients (at least 2,000) who underwent AS-OCT imaging between 2008 and 2020. AS-OCT images as well as demographic characteristics including age, gender, height, weight and body mass index (BMI) will be collected from electronic medical records (EMRs). The dataset of horizontal AS-OCT images will be split into training (80%), validation (10%), and test (10%) datasets, and a Vision Transformer (ViT) model will be built to predict demographics. Gradient-weighted Class Activation Mapping (Grad-CAM) will be used to visualize the regions of AS-OCT images that contributed to the model’s decisions. The accuracy, sensitivity, specificity, and area under the receiver operating characteristic (ROC) curve (AUC) will be applied to evaluate the model performance.

**Conclusion:**

This paper presents a study protocol for prediction of demographic characteristics from AS-OCT images of the eyeball using a deep learning model. The results of this study will aid clinicians in understanding and identifying age-related structural changes and other demographics-based structural differences.

**Trial registration:**

**Registration ID with open science framework:** 10.17605/OSF.IO/FQ46X.

## Introduction

Anterior segment optical coherence tomography (AS-OCT) is an imaging modality that provides non-contact, rapid, and high-resolution *in vivo* imaging of anterior segment structures [1]. With its technological advancement, AS-OCT is becoming more clinically useful for diagnosis, monitoring, and treatment of various ocular diseases, and also for preoperative evaluation (e.g. before refractive surgeries) [2–13]. For accurate diagnosis and disease-progression determination, however, it is crucial to establish the normal ocular structure, which is possible only based on a thorough understanding of structural changes and differences related to demographic characteristics.

Previous AS-OCT imaging studies have reported anatomic differences of anterior segment structures including the cornea, anterior chamber, iris, iridocorneal angle, and trabecular meshwork that correspond to demographic variables such as age, gender, height or weight [14–26]. In analyzing large amounts of imaging data, the recent introduction of deep learning to medicine, which is a form of representation learning whereby multiple processing layers automatically learn data representations with multiple levels of abstraction [27–29], has shown its potential additive value for the diagnosis and treatment of ocular diseases [30–37].

Although several studies have related structural differences of the anterior segment to demographic characteristics [14–26], each study analysis was limited to just a few specific structures, and their results, which were not verified, are difficult to apply to clinical practice. Furthermore, only a few studies have analyzed various demographic variables, necessitating investigation of structural differences using whole anterior segment structures with diverse demographic parameters. The aim of our study, accordingly, is to predict demographics from AS-OCT images using deep learning in normal eyes. Our findings will help clinicians to understand how anterior segment structures differ according to demographics and to identify the key structures that contribute to such differences.

## Materials and methods

### Study design and setting

**Fig 1:**
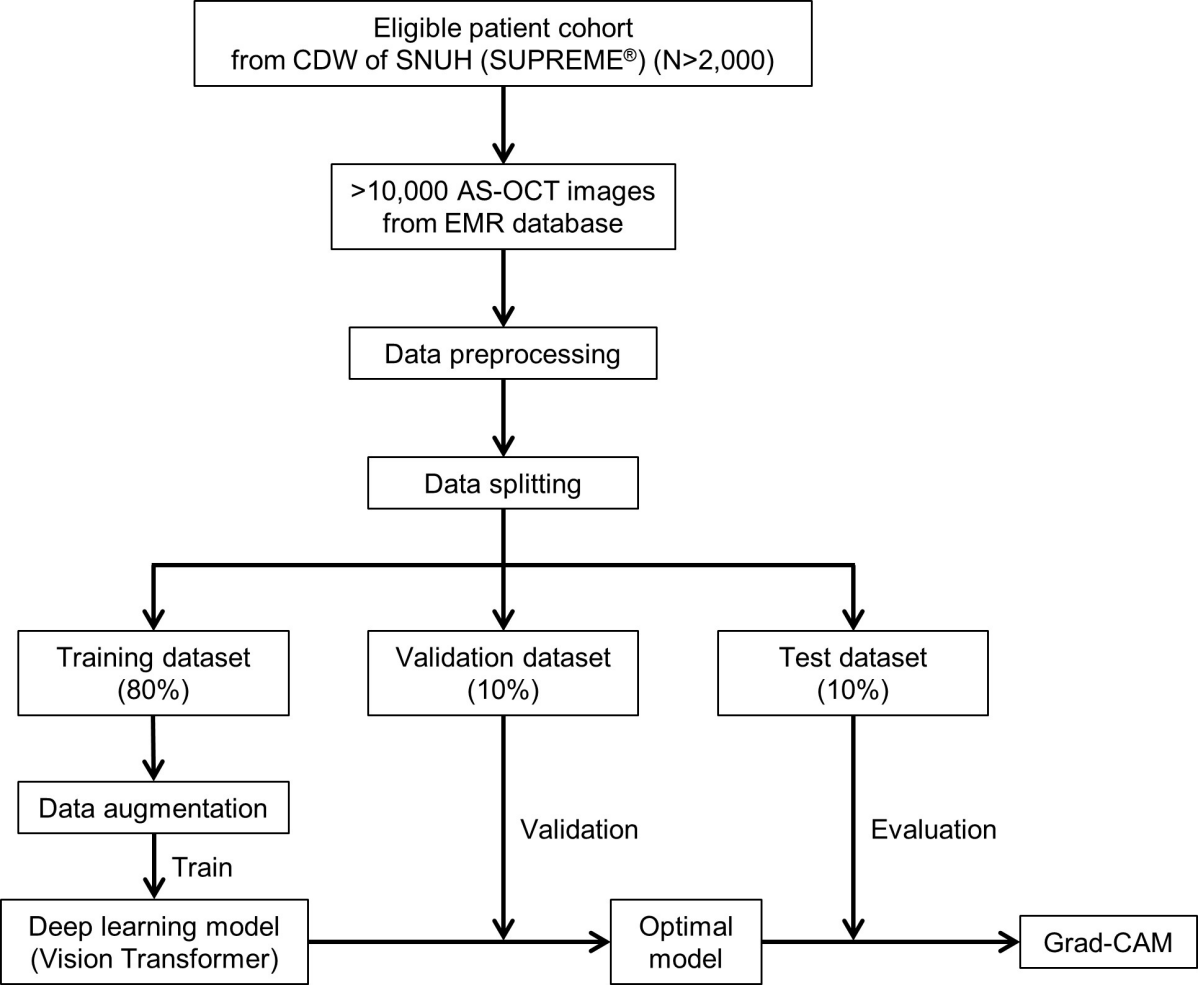
Study flow chart. Abbreviations: AS-OCT, anterior segment optical coherence tomography; CDW, clinical data warehouse; EMR, electronic medical record; Grad-CAM, Gradient-weighted Class Activation Mapping; SNUH, Seoul National University Hospital.

We will conduct a retrospective cross-sectional study including a large number of subjects (at least 2,000) from Seoul National University Hospital (SNUH) in South Korea, and the protocol has been registered in the Open Science Framework (https://osf.io/fq46x). A flow chart of our study is shown in Fig 1.

### Participants and data source

A list of eligible participants who meet the inputted search conditions (e.i. our study’s inclusion criteria (noted below)) on SUPREME^®^, a clinical data warehouse (CDW) of SNUH, will be obtained. AS-OCT (Visante; Carl Zeiss Meditec, Dublin, CA, USA) images and demographic data including age, gender, height, weight and body mass index (BMI) will be semi-automatically collected from an electronic medical record (EMR) database of BESTCare (ezCaretech, Seoul, Korea), a hospital information system of SNUH. As for the demographic data, we will obtain them through SUPREME^®^, which matches each patient’s data with his or her identification number.

### Inclusion criteria

Patients who underwent AS-OCT imaging between 2008 and 2020 at SNUH and for whom there are available height and weight measurements taken within 6 months of (before or after) the imaging will be included.

### Exclusion criteria

The exclusion criteria will be as follows: history of prior ocular surgery, laser treatment or trauma; any ocular or systemic diseases that could affect anterior segment structures, including corneal disease (e.g., corneal opacity, corneal dystrophy, keratoconus), iridocorneal angle abnormality (e.g., angle-closure glaucoma), or ocular inflammatory disease (e.g., uveitis); history of contact lens wear; any medications that could affect anterior segment structures such as the cornea, iridocorneal angle, or iris; any anterior segment abnormalities precluding visualization of the anterior segment structure (e.g., significant corneal opacity); any systemic diseases or therapies affecting height (e.g., Marfan syndrome) or weight (e.g., metabolic disease, cancer); AS-OCT images of poor quality or with artifacts.

### Sample size calculation

We expect that at least 10,000 AS-OCT images will be obtained from at least 2,000 patients. Since it is considered impossible to estimate the sample size required to train a deep learning model in advance, we will adjust it during the study based on the model’s performance.

### Study procedure

#### Data preparation

Each of the horizontal AS-OCT images will be split in half along the vertical midline and resized to 384 x 384 using bicubic interpolation. The entire dataset will be split into training, validation, and test datasets at a ratio of 8:1:1, and with the training dataset, the right-side of the split images will be flipped horizontally to align with the left-side images for data augmentation.

#### Deep learning model development

**Fig 2:**
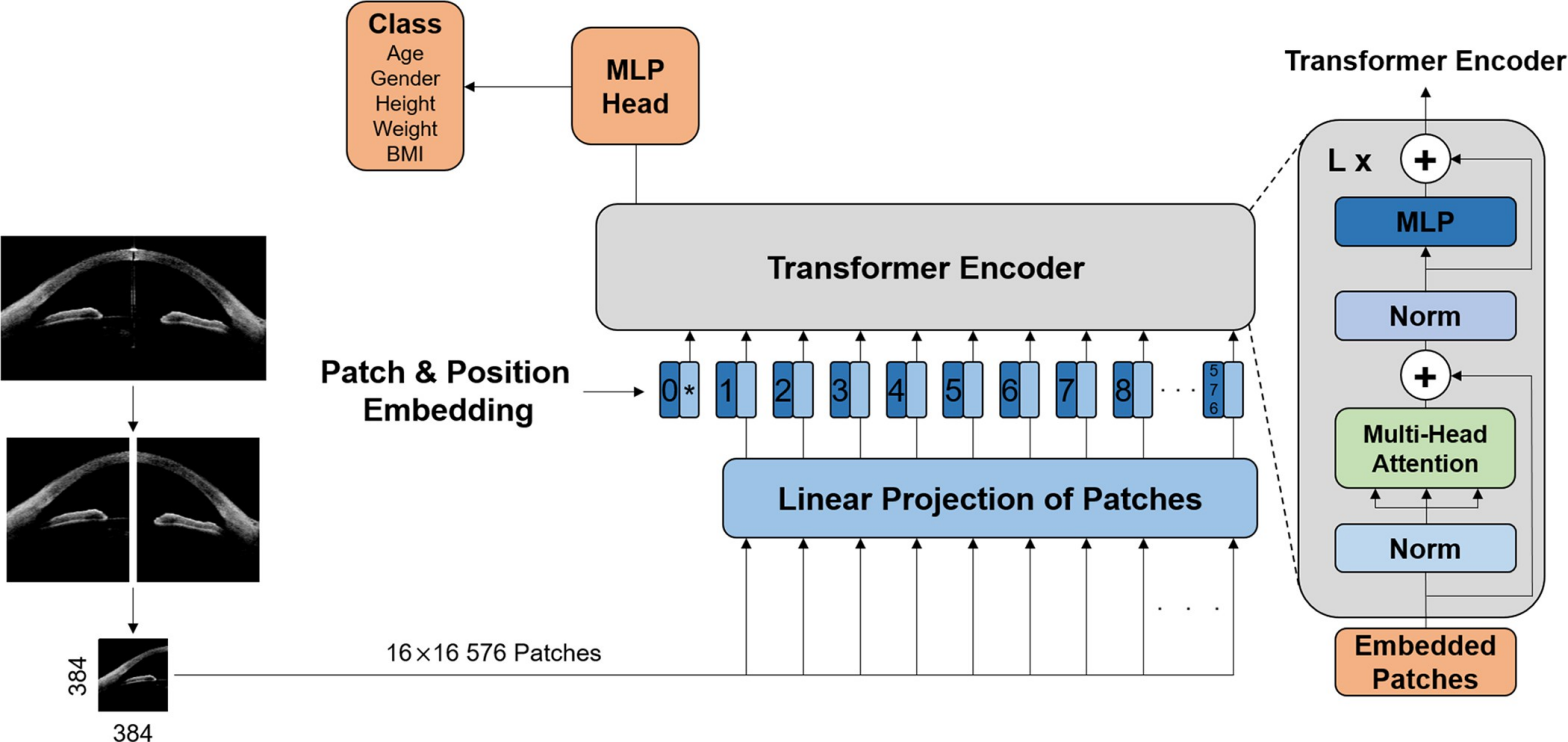
Vision transformer model. Abbreviations: BMI, body mass index; MLP, Multi-layer Perceptron.

For demographics prediction, we will utilize a Vision Transformer (ViT) model (available at https://github.com/lukemelas/PyTorch-Pretrained-ViT) that recently was applied for image classification and achieved state of the art, top-1 accuracy among the models classified with ImageNet [38]. Among the several ViT models, we will use the ViT-Base model (ViT-B/16), which will be pre-trained with ImageNet-21k and fine-tuned with ImageNet-1k. An overview of the ViT model is illustrated in Fig 2.

The 384 x 384 images will be fed into the network as multiple 16 x 16 patches (576 patches in total) that will be flattened to 1-dimensional vectors for inputs to the ViT encoder. The encoder consists of alternating layers of multi-head self-attention and Multi-layer Perceptron (MLP) blocks, and Layernorm (LN) and residual connections are respectively applied before and after every block [38]. The output of the transformer encoder will be passed through the MLP head, and the score value for each class will be obtained.

For each of the demographic characteristics, we will use the following cut-offs: ≤ 75 and > 75, ≤ 60, 60–75 and ≥ 75 years for age; ≤ 170 and > 170 cm for male height; ≤ 155 and > 155 cm for female height; < 70 and ≥ 70 kg for male weight; < 55 and ≥ 55 kg for female weight, and < 23, 23–25 and ≥ 25 kg/cm^2^ for BMI. Also, to adjust age, we will analyze other demographic characteristics including sex, height, weight and BMI using age-matched groups. As for age, height and weight, we will conduct the analysis dividing groups by sex to adjust it.

To visualize the regions of the AS-OCT images that contributed to the model’s decisions, Gradient-weighted Class Activation Mapping (Grad-CAM) [39] will be extracted from the first LN of the last block of the transformer encoder.

#### Hardware specifications

CPU: Intel(R) Xeon(R) Gold 5120 CPU @ 2.20 GHz.

GPU: Tesla V100 32GB x2.

#### Software specifications

Preprocessing: OpenCV 3.4.2.

Deep learning libraries: Pytorch 1.7.1, Python 3.7.

### Statistical methods and performance evaluation

To evaluate the performance of the deep learning model, the accuracy, sensitivity, and specificity will be calculated by the following equations. Also, the area under the receiver operating characteristic (ROC) curve (AUC), which is drawn by thresholding the output value of the designed network after normalizing (0~1) by the softmax function, will be calculated.


$$Accuracy=\frac{True\;Positive+True\;Negative}{Total\;number\;of\;subjects}$$

$$Sensitivity=\frac{True\;Positive}{True\;Positive+False\;Negative}$$

$$Specificity=\frac{True\;Negative}{True\;Negative+False\;Positive}$$

### Ethics and dissemination

This study was approved by the Institutional Review Board of SNUH (IRB No. H-2104-085-1212), and the collection and analysis of the data were permitted by the Big data Review Board (BRB) of SNUH. The study protocol followed the tenets of the Declaration of Helsinki. Informed consent will be waived due to the retrospective nature of the study. Study findings will be disseminated through publication in a peer-reviewed journal and presented at relevant conferences.

## Results

To evaluate the feasibility of the DL model, we performed a pilot study. For prediction of age, a total of 2,615 AS-OCT images were used in the analysis: 2,102 images (360 and 1,742 for age ≤ 65 and > 65 years, respectively) as a training dataset; 261 images (54 and 207 for age ≤ 65 and > 65 years, respectively) as a validation dataset, and 252 images (36 and 216 for age ≤ 65 and > 65 years, respectively) as a test dataset.

For classifying age ≤ 65 vs. > 65 years, the ViT model achieved an AUC of 0.816, which was lower than that of DenseNet121 convolutional neural network (CNN, AUC 0.843). With pre-training, however, the ViT model outperformed DenseNet121 CNN, achieving an AUC of 0.885 (Table 1).

**Table 1:** Performance of deep learning models for prediction of age ≤ 65 vs. > 65 years.

	AUC	Accuracy	Sensitivity	Specificity
DenseNet121 (CNN)	0.843	0.821	0.583	0.861
ViT (from scratch)	0.816	0.877	0.333	0.968
ViT (pre-trained)	0.885	0.885	0.556	0.940

Abbreviations: AUC, area under the receiver operating characteristic curve; CNN, convolutional neural network; ViT, Vision Transformer.

## Discussion

AS-OCT is, on the strength of its technical advancement, becoming an increasingly potent imaging modality for evaluation of various ocular diseases in the field of ophthalmology. In addition, the ViT, which is an extended, recent application of Transformer to computer vision inspired by its success in natural language processing, has attained excellent results in image classification and has shown its usefulness in ophthalmology as well [40, 41]. Indeed, our pilot study demonstrated a promising potential of ViT for prediction of age from AS-OCT images. Also, it showed that the pre-trained ViT outperformed CNN, which result being also supported by Dosovitskiy et al.’s study [38]. Combining the advantages of deep learning with those of AS-OCT, we expect that our study protocol will help to enhance the utilization of AS-OCT in clinical practice. Furthermore, our study is of great value in that it will enable obtaining hidden information on systemic factors from ocular imaging, as was also demonstrated in earlier studies [42–44].

The strengths of our study are its planned utilization of a deep learning method that will enable automated and fast analysis of massive, high-resolution AS-OCT imaging data from a large population. The limitations of the proposed research include potential selection bias due to its retrospective design, and its limited evaluation of structural differences among different ethnic groups. Further studies of prospective design that include diverse ethnic groups will further expand our knowledge and understanding of normal anterior segment structures.

## Conclusion

This paper presents a study protocol for prediction of demographic characteristics based on AS-OCT images of the eyeball using a deep learning model. The results of this study will help clinicians to better understand anterior segment structural changes and differences according to demographic variables in normal eyes, which should ultimately aid in evaluation and management of ocular diseases in clinical practice.
